# Placental contribution to the origins of sexual dimorphism in health and diseases: sex chromosomes and epigenetics

**DOI:** 10.1186/2042-6410-4-5

**Published:** 2013-03-21

**Authors:** Anne Gabory, Tessa J Roseboom, Tom Moore, Lorna G Moore, Claudine Junien

**Affiliations:** 1 INRA, UMR1198 Biologie du Développement et Reproduction, Jouy-en-Josas, F-78352, France; 2 Department of Obstetrics and Gynecology, Academic Medical Center, Amsterdam, the Netherlands; 3 Department of Clinical Epidemiology, Biostatistics and Bioinformatics, Academic Medical Center, Amsterdam, the Netherlands; 4 Department of Biochemistry, Biosciences Institute, University College Cork, College Road, Cork, Ireland; 5 Department of Obstetrics and Gynecology, University of Colorado Denver, Aurora, CO, USA; 6 UVSQ, Université Versailles Saint-Quentin en Yvelines, Guyancourt, France

**Keywords:** Epigenetics, Histone modifications, DNA methylation, Nutrition, DOHaD, Environment, Fetal programming, Sexual dimorphism

## Abstract

Sex differences occur in most non-communicable diseases, including metabolic diseases, hypertension, cardiovascular disease, psychiatric and neurological disorders and cancer. In many cases, the susceptibility to these diseases begins early in development. The observed differences between the sexes may result from genetic and hormonal differences and from differences in responses to and interactions with environmental factors, including infection, diet, drugs and stress. The placenta plays a key role in fetal growth and development and, as such, affects the fetal programming underlying subsequent adult health and accounts, in part for the developmental origin of health and disease (DOHaD). There is accumulating evidence to demonstrate the sex-specific relationships between diverse environmental influences on placental functions and the risk of disease later in life. As one of the few tissues easily collectable in humans, this organ may therefore be seen as an ideal system for studying how male and female placenta sense nutritional and other stresses, such as endocrine disruptors. Sex-specific regulatory pathways controlling sexually dimorphic characteristics in the various organs and the consequences of lifelong differences in sex hormone expression largely account for such responses. However, sex-specific changes in epigenetic marks are generated early after fertilization, thus before adrenal and gonad differentiation in the absence of sex hormones and in response to environmental conditions. Given the abundance of X-linked genes involved in placentogenesis, and the early unequal gene expression by the sex chromosomes between males and females, the role of X- and Y-chromosome-linked genes, and especially those involved in the peculiar placenta-specific epigenetics processes, giving rise to the unusual placenta epigenetic landscapes deserve particular attention. However, even with recent developments in this field, we still know little about the mechanisms underlying the early sex-specific epigenetic marks resulting in sex-biased gene expression of pathways and networks. As a critical messenger between the maternal environment and the fetus, the placenta may play a key role not only in buffering environmental effects transmitted by the mother but also in expressing and modulating effects due to preconceptional exposure of both the mother and the father to stressful conditions.

## Review

### Introduction

The recent and rapid worldwide increase in non-communicable diseases (NCDs) challenges the assumption that genetic factors are the primary contributors to such diseases [1]. There is compelling evidence based on numerous clinical observations and on experimental animal studies, that a new dimension, that of the “developmental origins of health and disease” (DOHaD) is at stake and therefore requires a paradigm shift [2]. Such studies are progressively revealing the role of early influences during gestation and lactation and, more recently, even during the preconceptional and childhood as well as adolescence periods on disease risk [3-11]. Exposure to various exogenous or/and endogenous changes during specific windows of developmental programming may affect the long-term health and susceptibility to NCDs of the offspring with a disparity between males and females in the timing of onset and severity of disease outcomes [12-16], often with a long latency [17].

As the interface between mother and fetus, the placenta plays a key role in fetal growth and development and, as such, affects the fetal programming underlying subsequent vulnerability in adulthood. Trophoblasts are the first cell lineage to differentiate during mammalian development. These cells mediate implantation and give rise to most of the extraembryonic tissues [18]. The placenta acquires nutrients and oxygen from the maternal circulation, returns waste products to the maternal circulation and prevents rejection of the semi-allogenic fetus. The placenta is also a major endocrine organ being responsible for synthesizing vast quantities of hormones and cytokines that have important effects on both maternal and fetal physiology [19-22]. As a gateway to the fetus the placenta is affected by numerous environmental factors including nutrient status and tissue oxygenation, which may modify epigenetic marks and gene expression within the placenta and therefore placental development and function [23-25]. Studies of rodents and large animals have shown placental development to be highly adaptable, with many means of compensating for poor nutritional conditions [26-30].

Sex differences in the rate of fetal growth have long been recognized [31]. The sex of the embryo affects the size of both the fetus and the placenta, together with the ability of the placenta to respond to adverse stimuli [27,32,33]. The placenta has traditionally been considered an asexual organ and therefore, many studies focusing on the placenta have not taken the sex of the embryo into account [33]. But given its extraembryonic origin, the placenta has a sex: that of the embryo it belongs to [33-35] and numerous DOHaD studies indicate that sex differences can originate early in development and in particular in the placenta [36]. Studies by Ishikawa et al. have clearly established an effect of sex chromosome « dosage » on placental size in mice, with XY placentas being significantly larger than XX placentas and that such differences are independent of androgen effects [37]. Although the possession of one X chromosome rather than two leads to an increase in placental size, the underlying mechanism is still to be determined [37].

In mice and cattle, accelerated development is already evident in XY blastocysts; cell division among male embryos occurs more rapidly than in female embryos [38] and, in humans, boys grow more rapidly than girls from the earliest stages of gestation [39]. These differences may start as early as the blastocyst stage in bovines: one third of genes showed sex differences in gene expression [40,41]. Gene expression analysis either for candidate genes or at the genome-wide level show that both the trajectories under basal conditions and those modulating responses differ between the sexes [15]. Analysis of genes involved in amino acid transport and metabolism identified sex differences both in average placental gene expression between male and female and in the relationships between placental gene expression and maternal factors [42]. Ontological analysis of such data suggests a higher global transcriptionnal level in females and greater protein metabolism levels in males. Specifically global glucose metabolism and pentose-phosphate pathway activity are twice and four times greater in bovine male vs. female blastocysts respectively, with similar metabolic differences being seen for human embryos at the same stages (for review [43]). At birth, placental weights and FPI (fetus-to-placenta weight ratio index, reflecting placental efficiency), tend to be greater in boys than girls [44]. These observations suggest that males may be both more responsive to growth promoting influences, and more susceptible to supply disturbances [44,45].

How could placental sex-specific functions under basal conditions, and sex-specific sensitivity to environmental conditions contribute to the differences in frequency, severity and age at onset of NCDs between the sexes? Unequal gene expression by the sex chromosomes between males and females play an important role even before implantation and the initiation of adrenal and gonad development. The burgeoning field of epigenetics provides credible molecular mechanisms to account for gene expression alterations that may persist in the long term. Owing to complex and programmable epigenetic processes, exposure to adverse environments during critical developmental windows can trigger long lasting influences on the cell’s-epigenome [46]. The resulting changes in epigenetic marks may alter cell fate decisions, the ensuing growth and development of tissues and organs, and subsequently be responsible for inadequate responses to later challenges such as an obesogenic environment in a sex-specific manner [15,47,48].

The aim of this review is to discuss the emerging knowledge on the sex-specific relationships between diverse environmental influences on placental functions and the risk of disease later in life. Given the abundance of X-linked genes involved in placentogenesis, and the early unequal gene expression by the sex chromosomes between males and females, this review focusses on the role of X- and Y-chromosome-linked genes, and especially on those involved in the peculiar placenta-specific epigenetics processes, giving rise to the unusual placenta epigenetic landscapes.

### Sex-specific outcomes of the effects of placental growth on fetal programming

**Figure 1:**
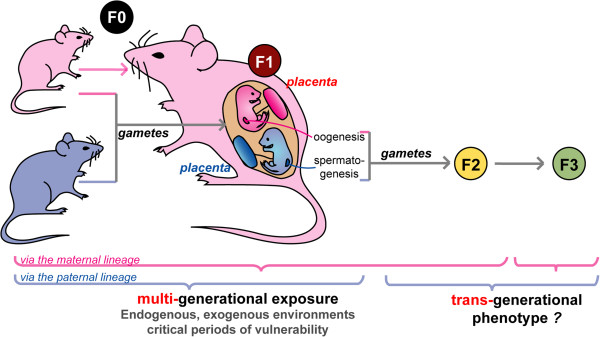
**Sex-specific transmission of exposure to environment to subsequent generations.** Environmental factors - including nutrition, psychosocial stress, toxins, endocrine disruptors, tobacco, alcohol, microbiota – impact individual (F0) epigenetic landscapes hence gene pathways and networks in ways that differ between the sexes. For example maternal and paternal preconceptional exposures can modify gamete quality and be transmitted to the subsequent (F1) generation. Additionally consequences of maternal F0 exposure during pregnancy (stress, metabolism, diet, hormonal changes…) can be transmitted from the maternal to the fetal compartment via the placenta in a sex-specific manner and affect F1 tissue development. Programming of somatic tissues can lead to changes in long-term health outcomes in the first generation. Moreover, primordial germ cells, which develop and undergo reprogramming during fetal development, can also be affected by F0 maternal environment and contribute genetic and epigenetic information to the F2 generation. Maternal and paternal lineages affect the transmission of such influences differently. In particular, multigenerational exposure on the maternal lineage can be seen in the F0, F1 and F2 generations, and transgenerational phenotype would be observed in F3, whereas on the paternal lineage multigenerational exposure concerns F0 and F1, and transgenerational phenotype in F2 and F3 generations.

As a critical messenger between the maternal environment and the fetus, the placenta may play a key role not only in buffering environmental effects transmitted by the mother but also in expressing and modulating effects due to preconceptional exposure of both the mother and the father to stressful conditions. Figure 1 shows how such influences may operate on the transmission of environmental influences to subsequent generation(s), and illustrate the central role of the placenta on the sex-specificity of these parent-of-origin effects. Support for the possibility of inter and transgenerational effects are also emerging, making it important to know the role played by the placenta and the possible maternal and or paternal epigenetic imprints carried by the gametes forming the zygote. Indeed, maternally or paternally transmitted non-erased epigenetic alterations of key developmental genes may perturb early trophoblast development in a sex-specific manner (Figure 1).

There is evidence to suggest that not only maternal mal- or undernutrition in the context of famines [20,49], maternal overnutrition, gestational diabetes or obesity, maternal stress or depression, but also environmental stressors such as drugs [50] and endocrine disruptors [51] are deleterious to the health of the offspring. Many of these factors have been shown to have the same range of defects and lead to the development of the metabolic syndrome [52-56], or mental health disorders in the offspring [57,58] with striking sex-specificity [15,59-63]. Two common complications of pregnancy, pre-eclampsia and asthma, have provided valuable insight into the way in which the feto-placental unit influences maternal physiology in a sex-specific manner. There is also growing evidence to suggest that some of these changes depend on the sex of the fetus [60,64,65]. In normal pregnancies, maternal microvascular vasodilatation, which is induced by placental corticotrophin-releasing hormone, is greater in pregnant women carrying male fetuses than in those carrying female fetuses. In pregnancies complicated by pre-eclampsia, microvascular vasodilatation in women carrying a male fetus is weaker than that in normotensive women carrying a male fetus, whereas no such difference is observed in women carrying female fetuses [64]. The human placenta adapts in a sexually dimorphic manner to chronic maternal asthma. In this situation, female fetal growth is limited, increasing the chances of survival, whereas male fetuses grow normally, this normal growth being associated with a poor outcome in cases of acute asthma exacerbation [33].

#### How unbalanced parental nutrition perturbs these differences

Placental growth has been shown to respond to maternal influences, including nutrition. There is evidence that the responses are different for the sexes. Studies among babies born around the time of the Dutch famine near the end of the Second World War (1944–1945) have provided insight into the effects of undernutrition on placental size and efficiency in humans, as well as regarding the existence of sex differences in these effects. Maternal undernutrition in early gestation resulted in a smaller placenta with the decrease in placental area being greater for boys than for girls. Famine also impaired placenta development even for pregnancies occurring after the famine had officially ended. Famine in mid to late gestation made the placenta less efficient as indicated by being born lighter than predicted from placental area, but more efficient when the famine occurred in early gestation or for conceptuses conceived after the famine had ended, since such babies were heavier than predicted [20]. In addition to the sexual dimorphism in the acute effects of undernutrition on placental size, the association between placental size and later health also appeared to differ between the sexes. In men, the association between placental size and later hypertension was completely reversed by famine exposure, while the associations were unaltered by famine in women [14].

Consistent with observations in humans, the restriction of placental function alters heart development in sheep fetuses, and small size at birth is associated with more components of metabolic syndrome in adult rams than in adult ewes [66,67]. Experimental and epidemiological studies in humans and animals also demonstrate an association between low or high FPI and impaired glucose tolerance, blood pressure and coronary heart disease [68].

The size and shape of the placenta are predictive of childhood blood pressure. Changes in the placentation process affecting implantation, the expansion of the chorionic surface in mid-gestation or the compensatory expansion of the chorionic surface in late gestation may affect blood pressure responses and the potential development of hypertension later in life. The adverse effects of small placental size may be compounded by those of poor maternal nutrition, whereas the area of the placenta may expand to compensate for fetal undernutrition in better-nourished mothers [69]. Changes in placental structure, activity or physiology may thus contribute to the programming of cardiovascular disease (CVD) in sex-specific ways [22,39,70]. For example hypertension in the male subjects in the Helsinki birth cohort born between 1934 and 1944 was associated with a long minor diameter of the placenta. Growth along this minor axis may be more sensitive to nutritional factors than growth along the major axis [71]. By contrast, hypertension in women was associated with a small placental area at birth, potentially indicating lower levels of nutrient delivery to the fetus. The greater dependence of boys on the diet of their mothers may enable them to make the best use of increases in food supply, but it also leaves them vulnerable to food shortages. This may be reflected in the tendency of men to have higher blood pressure and to die younger than women [72].

The effects of maternal undernutrition on placental growth and development have been studied in detail. However, fewer studies have focused on the potentially deleterious effects of maternal overnutrition or metabolic disturbances on the future health of the offspring, particularly as concerns the development of metabolic syndrome or the combination of obesity, type 2 diabetes (T2D) and CVD [53,73]. The embryo may also react to maternal overnutrition even before the placenta is formed - at the oocyte, zygote or blastocyst stage [74,75]. Moreover there are now convincing data showing that ancestral exposure to an environmental compound modifies the perception and response of the offspring to stress experienced during their own life history. While the effect of fetal sex on placental development and growth are known, there is relatively little known concerning sex differences in the context of overnutrition. Interestingly expression studies, although rare, do show a sex effect [27,61].

The Aberdeen Maternity and Neonatal Databank involving 55,105 pregnancies showed that maternal body mass index was positively associated with placental hypertrophy and birth weight but negatively associated with FPI, suggesting that being overweight or obese was associated with greater placental weight but lower placental efficiency [45]. In humans, placental weight and birth weight are lower in mothers with high carbohydrate intakes in early pregnancy. Low maternal intakes of dairy and meat proteins in late pregnancy are also associated with lower placenta weight and birth weight [76]. In mice, maternal obesity, T2D and a high-fat diet (HFD) during gestation increase adiposity and modify metabolism and blood pressure in adult offspring fed a control diet (CD), revealing a predisposition to the development of metabolic syndrome [77,78]. These findings suggest that impaired placental development under conditions of maternal overnutrition modifies fetal programming, resulting in impaired responses to diet in adulthood [22,27,71,79,80]. In pregnant mice fed a HFD during gestation, placental weight was higher and placental efficiency (FPI) lower, regardless of the sex of the fetus, without any gross changes in the areas or proportions of the labyrinth and junctional zone layers [81].

There have been few studies of paternal non-genetic effects on the health of the offspring in humans. However, epidemiological studies have suggested that a relationship between maternal grandmother's age and a major autistic trait, or paternal grandfathers access to food and rates of obesity and cardiovascular disease in subsequent generations in a sex-specific manner [82,83]. These aspects have been studied more thoroughly in rodents, in which clear evidence has been obtained for paternal effects on the phenotype and health of the offspring [84]. In particular paternal fasting before mating [85], paternal exposure to a HFD [8] or to a low-protein diet [6], and maternal caloric undernutrition during late gestation [7] all have been shown to alter metabolic function in the offspring. Additionally a human study involving 2947 singletons found a positive association between paternal weight and placental weight [86]. Thus, like maternal exposure, prior paternal exposure may have effects on placenta growth. However, to our knowledge, the effects of prior paternal exposure on placenta growth, size and shape have yet to be investigated, in order to elucidate mechanisms by which paternal influences, not just maternal ones, may be transmitted to the embryo, hence to the placenta.

#### Parental stress and behavior, neurobiology

Prenatal exposure to maternal stress, depression and pathogenic infections are associated with a higher risk for the development of neurodevelopmental disorders, including schizophrenia and autism [13,87]. Early childhood adversity has also been associated with earlier cancer incidence [11]. Clear differences between the sexes have been found in the programming of emotionality in the offspring and strategies for coping with stress, with the activational effects of testosterone producing females with male-like strategies in tests of passive coping, but with female-like behavior in tests of active coping [46].

Animal models of prenatal stress (psychological, behavioural, nutritional, or metabolic…) have identified major sex- and time-specific effects on the offspring. Maternal stress is associated with the dysregulation of stress pathways, a common feature in most neurodevelopmental disorders. Stress in early pregnancy has a significant sex-dependent effect on placental gene expression, modifying the fetal transport of key growth factors and nutrients [88]. Synthetic glucocorticoids affect the fetal programming of hypothalamic-pituitary-adrenal axis function and behavior [89]. However, high levels of the 11βHSD2 enzyme, which converts active glucocorticoids to an inactive metabolite in the placenta, protect the developing fetus from high maternal levels of this hormone [50,90]. Sex-specific differences in the cortisol stress response occur before birth, with much higher levels of cortisol output for male than for female fetuses [91]. Multigenerational programming in glucocorticoid-programmed rats is associated with effects on fetal and placental weight that are generation-specific and dependent on the parent of origin [92].

Recent reports have also highlighted the possibility of paternal transmission of stress-induced conditions, such as social defeat [93] and chronic and unpredictable postnatal maternal separation [94]. Behavioral adaptations that occur after the stress of chronic social defeat can be transmitted from the father to his male and female F1 progeny. The male offspring of defeated fathers also display increased baseline plasma levels of corticosterone and decreased levels of vascular endothelial growth factor [93]. Chronic and unpredictable postnatal maternal separation leads to perturbations in social abilities and serotonergic functions as well as traumatic experiences in early life [87]. The profile of DNA methylation is altered in the promoter region of several candidate genes in the germline of the separated males. Comparable changes in DNA methylation are also present in the brain of the offspring and are associated with altered gene expression [94]. This highlights the negative impact of early paternal stress on behavioral responses across generations and on the regulation of DNA methylation in the germline. However neither of these studies analyzed the effects on placentas of the subsequent generation(s).

#### Early life exposures to environmental toxicants, endocrine disruptors

In-utero and early-life exposures to environmental toxicants, ranging from heavy metals to endocrine-disrupting chemicals, affect adult metabolism, immune system function, neurodevelopment, and reproductive function. It is now evident that early-life exposures during the prenatal/fetal and postnatal period increase the risk for developing cardiovascular disease, diabetes, obesity, stroke, renal disease, osteoporosis, Alzheimer’s disease, and cancer [95]. For example chemical factors can behave as endocrine disruptors and as such perturb the developing endocrine and reproductive systems in a sex specific manner, either directly on somatic tissues of the exposed individual(s) and/or on their germline with the possibility of being transmitted to the next generation(s) through epigenetic mechanisms (Figure 1). Sex-specificity may also be generation-specific [92,96]. Thus disease susceptibility may reflect developmental exposures rather than simply exposure at or near the time of disease detection. Many of these compounds are lipid soluble and can accumulate in adipose tissue, with the possibility of being transferred across the placenta and fetal blood brain barrier. A number of studies in humans and rodents have demonstrated that gestational/perinatal exposure to either bisphenol A (BPA) or a common organophosphate pesticide interferes with several endocrine pathways and abrogates sexual dimorphism or shows altered sex differences in brain structure, or in heart respectively [97,98]. However while several studies considered the effects as a clear consequence of the transplacental deposition (reviewed in [99]), only one, to our knowledge, has examined the effects on placental gene expression, showing that BPA can alter miRNA expression in placental cells [100]. Thus investigating the effects of these compounds both on sexually dimorphic placental functions and on later health is of great interest.

### Mechanisms of unequal expression of X- and Y-chromosome-linked genes

#### Sex differences: sex hormones and/or sex chromosomes?

Increasing numbers of reports are challenging the traditional view regarding the influences of gonadal hormones and highlighting the potential roles for sex chromosomes (reviewed in [15,60,101,102]). Data from spotted hyena showed that the reduced expression of placenta aromatase may allow the hyena placenta to convert high circulating concentrations of androstenedione to testosterone, and could explain the virilization of the fetal external genitalia in female fetuses [103]. However, current data highlight a sexually dimorphic difference in placental function that may not be conferred by classical assumptions of sex steroid regulation. Testosterone may act in a sex specific manner in the human placenta and may be more potent in female placentas than males; however further investigations into the role of testosterone in placental function are required [33]. Nonetheless, unequal gene expression by the sex chromosomes has an impact much earlier, beginning at conception, and may set the context for events in later life (reviewed in [15,33,102,104]. Sex-linked genes and sex hormones may work together to yield similar differences in physiology between the sexes in brain. For instance, immune responses and cytokine production, or sex-linked genes like the androgen receptor, or Y-linked genes may exhibit sex differences because they can be influenced differently by steroid hormones (reviewed in [105]). Thus, unfavorable programming, whether immediately before conception or during gestation, may result in various defects potentially translated into differences in susceptibility to disease between males and females [8,15,33,60,72,81,106].

#### Early involvement of sex chromosomes in sex differences

Even before implantation and the initiation of adrenal and gonad development, transcriptional sexual dimorphism is present in various species that has consequences for developmental competence and adult health and disease [43]. For example, in bovine blastocysts, sex determines the expression levels of one-third of all actively expressed genes [107]. Sexual dimorphism has also been observed in embryonic cells isolated from mice at E10.5. These cells responded differently to dietary stressors even before the production of fetal sex hormones [108]. In the mouse, detailed studies on sex chromosomal contribution to placental growth have been reported [37]. The X chromosome has been implicated in causing several malformations of the placenta. About 30% of all trophoblast-expressed genes are on the X chromosome, and alterations in many different X-linked genes could account for similar phenotypes [109,110]. Due to paternal X inactivation in trophoblast cells, mutations in these X-linked genes manifest themselves in embryonic lethality upon maternal transmission of the mutant allele in the mouse. A role for the Y chromosome in placental dysplasia has also been demonstrated [111]. It is also well-established that male fetuses have a higher rate of perinatal complications attributed to placental dysfunction that may relate to the abundance of X-linked genes involved in placentogenesis [112].

#### Unequal dosage and compensation mechanisms between males (XY) and females (XX)

Mammals have a very complex, tightly controlled, and developmentally regulated process of dosage compensation between males (XY) and females (XX). Two main kinds of dosage compensation exist: the first being to avoid X hyperexpression in females by equalizing the expression of the X-linked genes via inactivation of one of the two X-chromosomes in females (XCI: X-chromosome inactivation) and the second leading to the balanced expression between X-linked and autosomal genes via transcriptional upregulation of the active X in both sexes, males and females. There are two forms of XCI—imprinted and random [113,114]. The incomplete, and unstable imprinted inactivation of the paternally inherited X-chromosome is observed in certain eutherians (for example, rodents) at pre implantation stages of embryonic development and is retained in the extraembryonic organs that derive from the fetus. Therefore, in mice, the paternal X chromosome is inactivated in the placenta [115]. In the cells that form the tissues of the embryo proper, the paternal X-chromosome is reactivated during implantation followed by a random inactivation of either the paternal or maternal X-chromosome [116]. The paternal imprint in the blastocyst trophectoderm and their derivates such as placenta seems to be unique to mice, not occurring in rabbits, bovines or humans where XCI occurs after the blastocyst stage [113].

However, not all X-linked genes are absolutely balanced. Several X-linked genes can escape XCI. More genes escape XCI in humans than in the mouse. While it has been estimated that 15 to 25% of the 1400 X-linked human genes escape XCI in humans, only 3% do so in the mouse [117-119]. There are also significant differences in terms of the distribution of « escape genes » in humans and mouse, with a random distribution along the mouse X chromosome, suggesting that escape is controlled at the level of individual genes rather than chromatin domains. This suggest that men and women may demonstrate greater sex differences in X linked gene expression than mice as a result of the large number of escape genes. In addition, the degree of escape, hence the expression levels from inactive X, can vary considerably between loci, ranging from 5% to >75% of active X levels [117]. Although there are no data on the laboratory mouse, it is interesting that in common voles, more genes were expressed on the inactive X chromosome in extraembryonic tissues than in somatic tissues [120]. Escape from XCI can vary between different tissues and/or individuals and the escape can also be developmentally regulated. In mice, silencing of some X-chromosomal regions occurs outside of the usual time window and escape from XCI can be highly lineage specific [113,116,121].

There are also additional control mechanisms to achieve balanced or unbalanced expression between the sexes. Some genes on the X-chromosome are imprinted: their expression is monoallelic, depending on the parental origin of the allele. Recently, three genes have been described as imprinted and expressed from the paternal X allele: *Fthl17*, *Rhox5* and *Bex1*. This monoallelic paternal expression is independent of XIC. Therefore, these genes are expressed predominantly in female [122].

#### Male-specific Y chromosome genes

In addition to unequal expression of X-linked genes, the small number of expressed genes present on the Y chromosome (and therefore only expressed in males) may be involved. In humans 29 genes are conserved in the pseudoautosomal regions (PARs) of the X- and Y-chromosomes [123]. The non-recombining, male-specific Y region contains about 27 protein-coding genes [124]. Some X/Y gene pairs have been retained on sex chromosomes and are referred to as paralogues. In the case of X/Y pairs, in contrast to humans, for which a number of X escapees do not have a Y paralogue, all known mouse escapees do have a Y paralogue [115,125]. Studies in mice and rats demonstrating sex differences in placental responses to changes in the maternal environment may thus indicate a role for these escaped genes, as the placentas of female fetuses may produce small differences in the amount of the corresponding proteins compared to amounts present in male fetuses. However, there are very few studies comparing levels of mRNA and proteins for escape vs. non-escape genes [101,126].

#### Placenta, brain and testis common evolutionary features?

The unique evolutionary pathway of the X- and Y-chromosomes has resulted in these chromosomes having highly atypical gene contents and activities [127]. The mapping of speciation genes has revealed one general rule: there is an apparent excess of sex and reproduction-related genes on the X-chromosome (reviewed in [128]). A preponderance of sex-and reproduction-related genes on the X chromosome has been shown repeatedly, but also mental retardation genes are more frequent on the X chromosome. Since the coordinate evolution of new characters is best attained when the same set of genes is redeployed, Wilda and co-workers suggested that new characters in the brain, testis and placenta are most responsible for human speciation [128].

Evolutionary constraints may thus be responsible for the presence of placental genes on the X chromosome that are co-expressed in brain and testis [109]. In human term placentas, Sood *et al.* have shown that many of the sex-correlated genes are located on the sex chromosomes, but that some are autosomal [129]. In addition, X- and Y-linked genes may modulate the expression of different sets of autosomal genes, leading to differences in physiological trajectories between males and females [15]. Thus, both the trajectories under basal conditions and those modulating responses differ between the sexes.

### Gene expression and epigenetic marks: mechanisms and dynamics

#### Sex-specific epigenetic marks modulate sex-specific gene expression

The study of the epigenetic marks and mechanisms underlying sex differences is in its infancy. The epigenetic landscape required for placenta development has been described [130]. The sex of the placenta and the environment have an influence on its epigenomes, and hence on the epigenomes of the developing fetus. In all adult tissues examined to date, including the gonads and brain, the expression of many genes is modulated in a sex-specific manner [15,131,132]. Chromatin structure and epigenetic marks differ between male and female samples in brain [133,134] The adult liver is the organ in which these aspects have been best characterized, with genome-wide DNaseI-hypersensitive sites and sex-specific gene expression detected [135-138]. However, even with recent developments in this field, we still know little about the mechanisms underlying the early sex-specific expression of genes and gene networks resulting from epigenetic regulation in the placenta.

Within the context of DOHaD, epigenetic marks, which respond to the environment, record the effects of the environment during development in a sex-specific manner [139]. Developmental alterations to epigenetic marks may induce long-term changes in gene expression, potentially leading to disease in later life [140,141]. Efforts are now being made to determine the contribution of epigenetics to the establishment and maintenance of sex differences. Most DOHaD studies have reported sex-specific transmission and/or effects, but very few have tackled the sex-specific epigenetic mechanisms involved, and especially in the placenta. In a recent review, Novakovic and Saffery suggested that DNA methylation profiling highlights the unique nature of the human placental epigenome for genomic imprinting and placenta-specific gene-associated methylation. Placental cell types have a pattern of genome methylation that is significantly different from that in somatic tissues, with low methylation at some, but not all, repetitive elements (reviewed in [47]).

Sexually dimorphic patterns of gene expression have recently been reported for individual genes in placentas from humans and rodents, potentially accounting for differences in the sensitivity of male and female fetuses to maternal diet (reviewed in [15]). Considering these expression studies, it is noteworthy that sex differences have been observed in the mRNA levels of housekeeping genes and of commonly used reference genes in human placenta, in a variety of mouse somatic and extra-embryonic tissues, as well as in the preimplantation blastocyst and blastocyst-derived embryonic stem cells [142,143]. Although this is not surprising given the importance of sexual dimorphism in every tissue examined so far, it underlines the difficulty in choosing appropriate reference genes. Few groups have studied global sexual dimorphism in the placenta with microarrays, focusing in particular on the impact of maternal diet, asthma or stress on placental gene expression, through systemic investigations of the relationship between diet and the expression of sexually dimorphic genes. These transcriptomic analyses showed that basal gene expression levels were sexually dimorphic in whole placentas [27,61,129]. Even fewer studies have investigated the associated epigenetic changes [61,81].

#### Sex-specific impact of environmental influences

The expression of key enzymes of the epigenetic machinery mapping to autosomes also appears to be sex-dependent, even at early stages [41,144]. Levels of *Dnmt1* are similar in male and female bovine embryos, but *Dnmt3a* and *Dnmt3b* are produced in smaller amounts in female embryos [41]. Levels of DNA methylation have been reported to be lower in XX ES cell lines than in XY or XO lines, and this hypomethylation is thought to be associated with lower levels of Dnmt3a and Dnmt3b [145]. In mouse placenta, global DNA methylation is also sexually dimorphic in animals fed the CD, with lower methylation levels in the placentas of male offspring than in those of female offspring at E15.5 stage. Under HFD, hypomethylation was observed only in the female placenta. Consistent with this observation, expression of the gene encoding the DNA methyltransferase cofactor *Dnmt3l* was downregulated in females only [61,81]. Clearly, further studies are needed to understand the direct effects of sex chromosomes and gonadal hormones on the regulation of genes controlling histone acetylation and methylation, coregulatory proteins and transient and stable DNA methylation patterns.

Expression analysis has also shown that maternal high-fat diet (HFD) affects mouse placental gene expression in a sexually dimorphic manner [61]. A HFD during gestation triggers the deregulation of clusters of imprinted genes. Sexual dimorphism and sensitivity to diet were observed for nine of 20 imprinted genes, from four clusters on mouse chromosomes 6, 7, 12 and 17. An analysis of CpG methylation in the differentially methylated region of the chromosome 17 cluster revealed sex- and diet-specific differential methylation of individual CpGs in two conspicuous subregions. Bioinformatic analysis suggested that these differentially methylated CpGs might lie within recognition elements or binding sites for transcription factors or factors involved in chromatin remodeling [81]. Gregg *et al*. recently reported sexually dimorphic genomic imprinting in the brain, with sex-specific imprinted genes found mostly in females [146,147]. Given the importance of genomic imprinting in the brain and placenta, this provides new clues for further investigations of sexual dimorphism in the placenta.

#### The special case of X/Y pairs of paralogues

In the same study, transcriptomic analysis showed that both basal gene expression and response to maternal HFD were sexually dimorphic in whole placentas. The differences between the sexes in the transcriptomic response to HFD were not only quantitative but also qualitative. The biological functions and networks of genes dysregulated differed markedly in sex-specific ways, with involvement of immune cells and uptake and metabolism of amino acids in females vs. the development and function of vascular system, and uptake and metabolism of glucose and fatty acids in males [61]. In this study, 11 genes displayed sexual dimorphism regardless of diet (control or HFD). Consistent with the key role of genes on the sex chromosomes, three of these genes were Y-specific, *Ddx3y, Eif2s3y* and *Kdm5d (Jarid1d)* and were more expressed in males, and three were X-specific, *Eif2s3x, Kdm5c (Jarid1c)* and *Ogt* and were more expressed in females. Interestingly, among these 6 X- and Y-linked genes, there were two paralogue pairs: *Eif2s3x/y* and *Kdm5c/5d*[61]. Of particular interest are the X-linked genes that encode enzymes of the epigenetic machinery and transcription factors: *Kmt1a* (*Suv39h1), Jpx, Xist, Kdm6b (Jmjd3), Kdm5c, Eif2s3x, Kdm6a (Utx), Ddx3x,* are on the X chromosome, as well as the corresponding paralogues for the latter ones, *Kdm5d, Eif2s3y, Uty,* and *Ddx3y* that are on the Y chromosome. Sex-specific differences in expression of the histone demethylases *Utx/Uty* and *Kdm5c* have been observed in mouse brain and neurons [148,149]. Other studies have reported the male-specific expression of Y-linked genes — *Ddx3y, Eif2s3y* and *Kdm5d* — in mouse hearts and human myocardium [150]. In mouse brain, Reinius and coworkers recently identified 4 female-biased long non-coding RNAs (lncRNAs) associated with protein-coding genes that escape X-inactivation, the *Ddx3x/Kdm6a* cluster*, Eif2s3x, 2610029G223Rik,* and *Kdm5c*[151]. Given that placenta, brain and testis could share common mechanisms involving X-linked genes [128], these lncRNAs might also be implicated in placental development or function. Moreover, these mouse escapees from X-inactivation also have a paralogue on the Y-chromosome. According to the authors, these lncRNAs might also escape X-inactivation [151]. It would thus be interesting to investigate how these three mechanisms (escaping X inactivation, X/Y paralogues and lncRNA) participate in sexual dimorphism.

**Figure 2:**
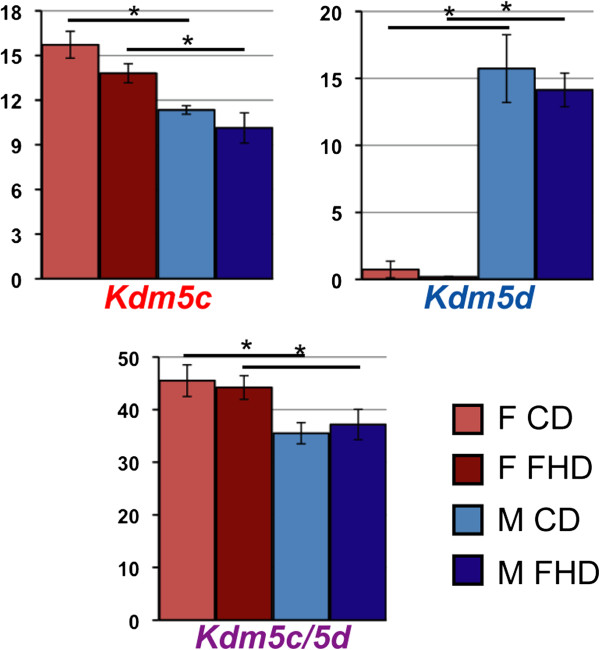
**Sex specific expression of the X/Y paralogues *Kdm5c* and *Kdm5d*.** Three PCR primer pairs have been designed for recognizing specifically either *Kdm5c* or *Kdm5d* cDNA and for recognizing both *Kdm5c*/*5d* cDNA. Their expression was studied in male and female placentas in pregnant female mice fed either a control diet (CD) or a high-fat diet (HFD) from E0.5 to sacrifice at E15.5 stage. *Kdm5c* expression is higher in females (pink bars) than males (blue bars), and *Kdm5d* is expressed only in males, regardless of maternal diet. The *Kdm5c*/*5d* PCR shows that the combined expression of *Kdm5d* and *Kdm5c* expression in males is not of equivalent magnitude as the expression of *Kdm5c* from both alleles in females.

The proteins encoded by Y-linked genes may or may not have the same functions, the same target sequences or the same pattern of expression, according to age or tissue, as their X paralogue. In our study, in placenta of HFD fed mouse mothers, the Y- and X-linked histone demethylase paralogue genes *Kdm5c* and *Kdm5d* were sexually dimorphic. In another report, in mouse brain, expression of the Y version of the gene in male mice did not compensate for the dosage imbalance between the two sexes in the expression of their X homologs escaping X-inactivation. Figure 2 shows that, in placentas from mothers fed a control or high-fat diet, the Y-linked *Kdm5d* gene expression in males is not able to compensate the expression of *Kdm5c,* its X-linked paralogue escaping XIC, in females [61]. Thus the epigenetic enzymes produced by these two genes could mark the epigenome in a sex-specific manner, both at the quantitive and qualitative levels [152].

## Conclusion

The DOHaD concept is consistent with the possibility that environmental influences can affect the development of sex differences early in development and in particular in the placenta, sculpting its epigenomes, and hence the epigenomes of the developing fetus [36]. But where, how, and when sex differences begin in the placenta and how they contribute to sex-specific responses of somatic tissues later in life is still poorly understood. The sex of the embryo affects the size of both the fetus and the placenta, and the ability of the placenta to respond to adverse stimuli [27,32,33]. Female and male placentas have different routes to maximize fitness and therefore the two sexes have different optimal transcriptomes that may affect fetal growth and later disease susceptibility or health trajectory [61]. Differences in how male and female placentas cope with stressful conditions indicate that this tissue should also be taken into account if we want to understand how it contributes to sexual dimorphism later in life. The placenta may therefore be seen as an ideal system to study the sensing, by the fetus, of stresses, starvation, endocrine disruption and obesity-prone diets or lifestyles, in a sex-specific manner [51,88].

Several critical issues remain to be addressed for unravelling the sexually dimorphic nature of programming in utero. We still know little about the mechanisms underlying the early sex-specific expression of genes and gene networks resulting from epigenetic regulation in the placenta. Elucidation of the biological basis of differences in male and female development will improve our understanding of the respective contributions of hormones, X- and Y-linked genes, autosomal genes, and their possible synergistic or antagonistic interactions [12,105,138]. An understanding of these factors and of the sex-specific genetic and epigenetic architecture of human disease might also reveal the existence of sex-specific protective mechanisms that could be exploited in novel treatments [153]. Thus if we are to use the placenta as an indicator of what occurred in utero, it is crucial to understand how, in addition to sex-specific differences in the endocrine and immune systems [154,155], sex-specific genetic architecture [156] also influences placental growth and specific functions [112,157], both under normal conditions or severe placental dysfunction [63,158]. Finally, unravelling the epigenetic marks and mechanisms underlying these sex differences in physiological trajectories and in response to environmental changes represents a major health challenge.

These findings highlight the importance of studying both sexes in epidemiological protocols and dietary interventions. Where possible, effects should be investigated in a sex-specific manner in order to provide solid scientific evidence for sex-specific interventions and recommendations. The striking sexual dimorphism for programming trajectories necessitates a considerable revision of current dietary intervention protocols. The identification of sex-specific explanations of the responses and adaptation of males and females to dietary quality, quantity and other environmental factors should help physicians and patients anticipate the major challenges likely to occur during the patient’s lifetime. In that context, placental analyses could be used to identify children at risk of adverse programming.

Owing to the flexibility of epigenetic processes, the DOHaD and their underlying epigenetic mechanisms offer a new possibility to envisage a comprehensive and evidence-based plan of nutritional, behavioral, and socio-economical recommendations to apply new cost-effective preventive actions against NCDs, in a sex-specific manner [159-161].

## Abbreviations

CD: Control diet; CVD: Cardiovascular disease; Dnmt: DNA methyltransferase; DOHAD: Developmental origins of health and disease; FPI: Fetus-to-placenta weight ratio index; HFD: High-fat diet; Kdm: Lysine-specific histone demethylase; Kmt: Lysine-specific histone methyltransferase; lncRNA: Long non-coding RNA; NCD: Non-communicable disease; T2D: Type 2 diabetes; XCI: X-chromosome inactivation.

## Competing interests

The authors declare that they have no competing interests.

## Authors’ contributions

CJ edited this manuscript based on contributions from the other authors. All authors read and approved the final manuscript.
